# Supplementary material for: An AI-based chatbot to support health-related social needs among pediatric primary care population: Protocol for a pilot randomized controlled trial
Source: PLoS One. 2026 Apr 20;21(4):e0337868. doi: 10.1371/journal.pone.0337868 (PMC13094993; doi:10.1371/journal.pone.0337868)
Supplement: S1 Table — (DOCX) [file pone.0337868.s001.docx]

# **Supporting information Table 1. The details of study endpoints**

| **Outcome Domain** | **Instrument / Definition** | **Scale & Range** | **Direction** | **Timepoint(s)** |
| --- | --- | --- | --- | --- |
| **Retention Rate** | Proportion of randomized caregivers completing follow-up assessments | Proportion (0-100%) | Higher = better feasibility | 1, 3, and 6 months |
| **Recruitment Feasibility** | Enrollment rate (enrolled / eligible approached) | Proportion | Higher = better feasibility | Continuous monitoring; summarized at end of recruitment |
| **Survey Completion Feasibility** | Completion rates at each follow-up | Proportion | Higher = better feasibility | 1, 3, and 6 months |
| **Intervention Feasibility (Caregivers)** | Feasibility of Intervention Measure (FIM) | Per instrument manual | Higher = greater feasibility | 3 and 6 months (intervention arm) |
| **Acceptability (Caregivers)** | Website Evaluation Questionnaire (WEQ) | Per instrument manual | Higher = greater acceptability | 1 and 3 months (intervention arm) |
| **Usability (Caregivers)** | System Usability Scale (SUS) | 0-100 | Higher = better usability | 1 and 3 months (intervention arm) |
| **Caregiver Comprehension** | Patient Comprehension Questionnaire (PCQ) | Per instrument manual | Higher = better comprehension | 1 and 3 months (intervention arm) |
| **Caregiver Stress** | Validated stress measure | Per instrument manual | Higher = greater stress | Baseline, 3, and 6 months |
| **Caregiver Self-Efficacy** | Validated self-efficacy scale | Per instrument manual | Higher = greater self-efficacy | Baseline, 3, and 6 months |
| **Satisfaction with Resource Access** | Validated satisfaction measure | Per instrument manual | Higher = greater satisfaction | 6 months |
| **Caregiver & Child Quality of Life (Proxy)** | Validated QoL measure | Per instrument manual | Higher = better QoL | Baseline, 3, and 6 months |
| **HRSN Burden** | Standardized HRSN screener (count or categorical per instrument) | Count or categorical | Higher = greater unmet need | 3 and 6 months |
| **Caregiver Study Burden** | Participant Burden Assessment (PeRBA) | Per instrument manual | Higher = more favorable (lower perceived burden) | 1, 3, and 6 months |
| **Chatbot Engagement (Intervention Only)** | System logs (minutes used, logins, session duration, screenings completed, resources accessed) | Continuous counts | Higher = greater engagement | Continuous over 6 months; summarized at 6 months |
| **Provider Workflow Integration** | Workflow Integration Expectancy (WIE) | Per instrument manual | Higher = greater integration expectancy | 1 and 6 months |
| **Provider Feasibility** | Feasibility of Intervention Measure (FIM) | Per instrument manual | Higher = greater feasibility | 6 months |
| **Provider Usability** | System Usability Scale (SUS) | 0–100 | Higher = better usability | Baseline |
| **EHR-Derived Utilization** | HRSN documentation/referrals; well-child adherence; missed appointments; ED visits | Counts / binary events | Lower ED/missed visits = favorable | 3 and 6 months |
| **Estimated Healthcare Costs** | Billing and visit data abstraction | Continuous cost estimates | Lower cost = favorable | 6 months |
